# No relationship between male pubertal timing and depression – new insights from epidemiology and Mendelian randomization

**DOI:** 10.1017/S0033291724000060

**Published:** 2024-07

**Authors:** Raphael Hirtz, Corinna Grasemann, Heike Hölling, Björn-Hergen von Holt, Nicola Albers, Anke Hinney, Johannes Hebebrand, Triinu Peters

**Affiliations:** 1 Department of Pediatrics, Division of Rare Diseases, and CeSER, Ruhr-University Bochum, Alexandrinenstr. 5, 44791 Bochum, Germany; 2 Division of Pediatric Endocrinology and Diabetology, Department of Pediatrics II, University Hospital Essen, University of Duisburg-Essen, Hufelandstr. 55, 40211 Essen, Germany; 3 Helios University Medical Centre Wuppertal – Children's Hospital, Witten/Herdecke University, Wuppertal, Germany; 4 Department of Epidemiology and Health Monitoring, Robert Koch Institute, Berlin, Germany; 5 Institut für Medizinische Biometrie und Statistik, Universität zu Lübeck, Universitätsklinikum Schleswig-Holstein, 23562 Lübeck, Germany; 6 Department of Child and Adolescent Psychiatry, Psychosomatics and Psychotherapy, University Hospital Essen, University of Duisburg-Essen, Virchowstr. 174, 45147 Essen, Germany; 7 Center for Translational Neuro- and Behavioral Sciences, University Hospital Essen, University of Duisburg-Essen, Essen, Germany

**Keywords:** depression, epidemiology, male, Mendelian randomization, puberty

## Abstract

**Background:**

In males, the relationship between pubertal timing and depression is understudied and less consistent than in females, likely for reasons of unmeasured confounding. To clarify this relationship, a combined epidemiological and genetic approach was chosen to exploit the methodological advantages of both approaches.

**Methods:**

Data from 2026 males from a nationwide, representative study were used to investigate the non-/linear relationship between pubertal timing defined by the age at voice break and depression, considering a multitude of potential confounders and their interactions with pubertal timing. This analysis was complemented by Mendelian randomization (MR), which is robust to inferential problems inherent to epidemiological studies. We used 71 single nucleotide polymorphisms related to pubertal timing in males as instrumental variable to clarify its causal relationship with depression based on data from 807 553 individuals (246 363 cases and 561 190 controls) by univariable and multivariable MR, including BMI as pleiotropic phenotype.

**Results:**

Univariable MR indicated a causal effect of pubertal timing on depression risk (inverse-variance weighted: OR 0.93, 95%-CI [0.87–0.99)], *p* = 0.03). However, this was not confirmed by multivariable MR (inverse-variance weighted: OR 0.95, 95%-CI [0.88–1.02)], *p* = 0.13), consistent with the epidemiological approach (OR 1.01, 95%-CI [0.81–1.26], *p* = 0.93). Instead, the multivariable MR study indicated a causal relationship of BMI with depression by two of three methods.

**Conclusions:**

Pubertal timing is not related to MDD risk in males.

## Introduction

Major depressive disorder (MDD) is a debilitating psychiatric disorder and is expected to be the leading cause of disease burden worldwide in 2030 by the WHO (Doherty, Egan, & Dinneen, 2021). Childhood and adolescent psychopathology, including MDD, relates to poor mental health and social functioning in later life (Fergusson, Boden, & Horwood, 2007), implicating the need to identify children and adolescents at risk for early prevention and intervention (Bevan Jones et al., 2018). While the prevalence of MDD rises during puberty and is higher in adolescent girls than boys (Mojtabai, Olfson, & Han, 2016), also the 12-month prevalence of MDD in adolescent boys in western countries ranges between 5.4% (Lu, 2019) and 5.7% (Mojtabai et al., 2016) and cumulatively amounts to 13.6% between 12 and 17 years of age (Breslau et al., 2017).

However, the relationship between puberty and MDD is less consistent in boys than in girls (Hamlat, Stange, Abramson, & Alloy, 2014; Wang, Lin, Leung, & Schooling, 2016), likely for reasons of unmeasured confounding (Wang et al., 2016). Recently, Stumper and Alloy (2023) summarized a large number of potential confounders in this relationship. In particular, aspects associated with body weight, including body mass index (BMI) and body image perception, have been identified as confounding factors. Alongside, stressful and adverse life events, parental and social support, and socioeconomic status (SES) have been recognized as confounders (Lorant et al., 2003; Oelkers et al., 2020; Stumper & Alloy, 2023). Unlike the thorough analysis of the relationship between puberty and MDD in girls, some studies in boys did not account for confounders (Angold, Costello, & Worthman, 1998; Hamlat, McCormick, Young, & Hankin, 2020), and many only considered a limited subset.

Physical changes linked to puberty have been thoroughly analyzed, particularly in relation to pubertal timing (Hirtz et al., 2022a). Pubertal timing refers to the time of onset of pubertal development or the occurrence of important maturational events, including the age at voice break in males (Hirtz et al., 2022a). In women, we could recently show that an earlier age at menarche, a commonly used milestone to define pubertal timing, is related to an increased MDD risk in the general population (Hirtz et al., 2022b) as well as the severity of depressive symptoms in a clinical cohort of adolescent females (Hirtz et al., 2022a).

To consider the methodological limitations of epidemiological studies in the analysis of the relationship between male pubertal timing (MPT) and MDD, the present study utilizes Mendelian randomization (MR). As previously discussed (Hirtz et al., 2022b), MR uses genetic markers to draw causal conclusions on the association between an exposure (e.g. MPT) and an outcome (e.g. MDD) of interest by exploiting that genotypes are not generally associated with confounders in the population and are randomly assigned at conception, analogous to randomization in clinical trials. Moreover, since the individual genotype is determined upon conception and cannot be modified by the outcome of interest, MR is robust to reverse causation. Usually, single nucleotide polymorphisms (SNPs) derived from large-scale genome-wide association studies (GWASs) are used as instrumental variable (IV) to study the relationship between exposure and outcome. So-called horizontal pleiotropy poses a risk to the validity of MR results. This phenomenon denotes effects of the IV on the outcome (depression) via pathways other than the exposure (MPT). Previously, BMI has been identified as a potential pathway to horizontal pleiotropy when using MPT as an exposure: SNPs associated with pubertal timing are related to BMI (Busch, Hojgaard, Hagen, & Teilmann, 2020; Day et al., 2017), and a higher BMI is associated with an increased risk for depression (Casanova et al., 2021). Therefore, the analysis of the relationship between MPT and MDD needs to address pleiotropy by multivariable MR incorporating BMI.

However, two-sample MR studies that rely on summary statistics from independent GWAS on the exposure and the outcome (Burgess & Thompson, 2021) are often limited by studying the relationship of interest based on the statistical, usually linear model used in the GWAS to define the IV. Thus, it is not possible to study interactions between the exposure and other phenotypes in multivariable MR. Moreover, MR studies are not suitable for estimating effect sizes for specific developmental episodes, as significant findings represent the average effect of an exposure on the outcome over the life course (Burgess & Thompson, 2021). Thus, this approach is complemented by a large-scale, representative study of adolescents and young adults, also including information on the above-mentioned confounders in the relationship between pubertal timing and MDD.

Using this methodological approach, combing the methodological advantages of each strategy, the results of the present study may have implications at the population level to guide prevention measures that address the timing of puberty in boys. At the individual level, MPT may be considered part of a prospective risk score to determine the likelihood of MDD if identified as an important contributing factor.

## Epidemiological analysis

### Participants

As previously described (Hirtz, Holling, & Grasemann, 2022c), ‘The German Health Interview and Examination Survey for Children and Adolescents' (KiGGS) is a nationally representative longitudinal study on the health status of children and young people in Germany. Since the baseline study (2003–2006, *N* = 17,640, age range 0 to 17 years), two follow-ups have been completed: KiGGS wave 1 between 2009 and 2012 (*N* = 11,992, age range 11 to 24 years) and KiGGS wave 2 between 2014 and 2017 (*N* = 10,853, age range 18 to 31 years); 3,775 respondents did not take part in any follow-up assessment). Further details on the study design, sampling strategy, and study protocol have been described in detail elsewhere (Mauz et al., 2020) and are summarized in the online Supplemental Material 1 (SM1 – Supplementary Methods 1).

The KiGGS study was approved by the ethics committee of the Charité Berlin (baseline and wave 1) and the ethics committee of the Hannover Medical School (wave 2). Written informed consent was obtained from parents as well as from children aged 14 years and older.

**Figure 1.**
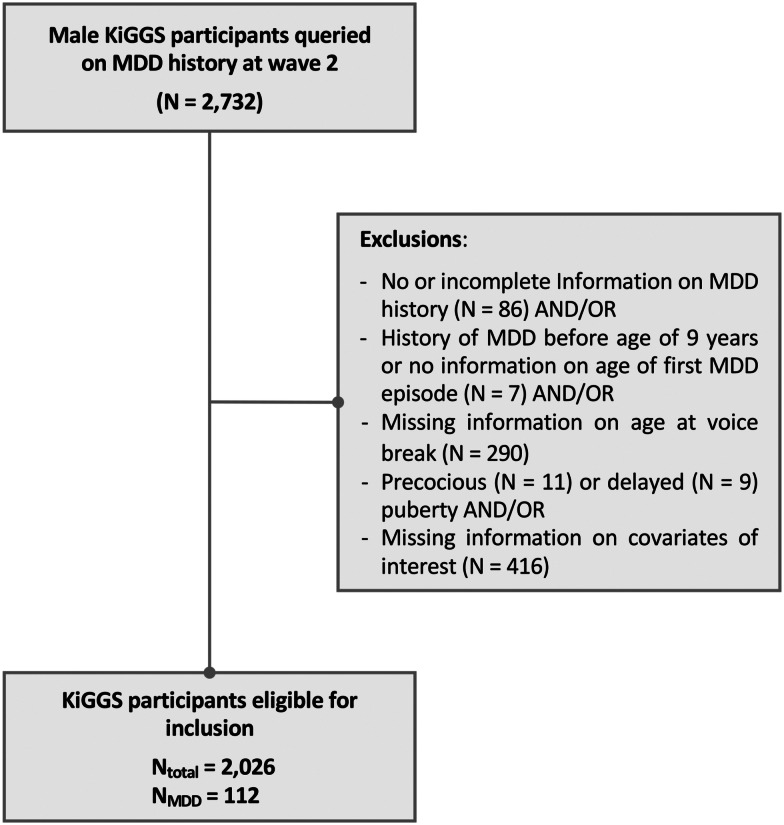
Study design flowchart. MDD, major depressive disorder. *Note*: Some participants had missing information on more than more variable.

For the present study, data from the baseline study and wave 2 were used. Male participants who were queried on MDD history were considered for analysis (*N* = 2,732). Participants with incomplete information on MDD history, a history of MDD before 9 years of age (i.e. before pubertal onset is expected in boys), precocious or delayed puberty, and missing information on pubertal development or any confounder of interest were excluded (Fig. 1).

### Questionnaires and interviews

Participants aged 11 years and older and parents of minor participants completed self-administered, standardized questionnaires. Among others, the questionnaires addressed physical and mental health as well as their determinants, including information on SES, social support, self-efficacy, and childhood trauma by standardized questionnaires (details on the questionnaires are provided in the online Supplemental Methods 1). Moreover, participants were queried on their subjective body image (much too thin, slightly too thin, exactly the right weight, too obese, or much too obese) and on adverse childhood experiences (own severe disease or accident and death; severe disease or accident of a close person).

Parents of minors and young adults (⩾ 18 years) additionally completed a computer-assisted personal interview (CAPI) conducted by a specially trained study physician (Mauz et al., 2020). Beginning with wave 2, the CAPI also covered the participants' mental health history, including the age of onset of mental health conditions (in total years), regarding selected disorders diagnosed by a physician (general practitioner, psychiatrist, neurologist) or psychologist.

### Male pubertal timing and physical examination

MPT was defined by age at voice break, considering its high correlation with other maturational pubertal events (Busch et al., 2019) and for consistency with the GWAS underlying the MR analysis. MPT was determined at wave 2 by retrospectively asking participants about the age at which their voice started to break (in total years) (Mauz et al., 2020). Based on this information, the total sample of male participants (*N* = 3,747) was used to define precocious (mean age at voice break ⩽ −2.5 SDS [0.4th percentile]) and delayed puberty (⩾ +2.5 SDS [99.6th percentile]) (Pathomvanich, Merke, & Chrousos, 2000).

Further details regarding anthropometric measures in the psychiatric sample and KiGGS participants are outlined in the online SM1 – Supplementary Methods 2.

### Statistical analysis – significance, effect size, and power

The results regarding the relationship between MPT and MDD were assessed by two-tailed testing and considered significant at *p* < 0.05. The results regarding the analysis of covariates, including those investigating interactions with MPT, were FDR-corrected for multiple comparisons at *q* < 0.05. The analysis of sociodemographic data and the pattern of missingness, including results from multiple imputation (see online SM1 – Supplementary Methods 3), were considered exploratory and not corrected for multiple testing.

The effect size of significant findings is reported as Cohen's *d* (*d* [small 0.31 ⩽ *d* ⩽ 0.49, medium 0.50 ⩽ *d* ⩽ 0.79, large ⩾ 0.8]). Post hoc power analyses were conducted with GPower 3.1.9.7 (Faul, Erdfelder, Lang, & Buchner, 2007), assuming a prevalence of MDD of 5%, *α* = 0.05, borderline small effect size (*d* = 0.2) per change in 1 s.d. of the predictor, and *R*^2^ of the predictor with other variables = 0.04.

### Logistic regression analysis

Two logistic regression models assessed the relationship between MPT (age at voice break in total years – independent variable) and the risk for MDD (dependent variable). The first model was unadjusted, the second model accounted for information on confounders related to MPT and MDD outlined in the introduction (BMI, SES, subjective body image, social support, self-efficacy, CTQ subscales emotional and physical abuse, and the sum score of childhood adverse events; details on the assessment of confounders are provided in online SM1 – Supplementary Methods 4) recorded at wave 2 (for details on testing the assumptions of logistic regression, see online SM1 – Supplementary Methods 5).

### Confounders – moderation and mediation analysis

Since confounders in the relationship between pubertal timing and MDD have the potential to act as moderators and/or mediators (Stumper & Alloy, 2023), a third logistic regression model was specified. This model, otherwise identical to model 2, additionally considered interaction terms between MPT and the above outlined confounders.

In case of a significant relationship between MPT and MDD demonstrated by either of the three models, a subsequent mediation analysis was intended to determine whether the observed relationship was driven by one the aforementioned confounders.

## Mendelian randomization

### Univariate MR analysis

In an MR study, three main assumptions must be met (Haycock et al., 2016): (1) the genetic instrument must have a strong association with the exposure, (2) the genetic instrument is independent of potential confounders in the relationship between the exposure and the outcome, and (3) the outcome is associated with the genetic instrument only through the effect of the exposure (Haycock et al., 2016).

The first assumption can be tested by the *F* statistic, calculated as (Beta/s.e.)^2^ for each SNP defining the IV for MPT. The *F* statistic for the total IV including all SNPs was calculated considering the proportion of the explained variance by the IV (*h*^2^_SNP_) and the sample size of the outcome (Burgess & Thompson, 2017). An *F* > 10 indicates that no bias due to a weak IV is present (Haycock et al., 2016). The second assumption is unlikely to be violated in the MR context (Greco, Minelli, Sheehan, & Thompson, 2015), as genetic variants are fixed at conception and cannot be influenced by confounding factors of the risk factor-outcome associations (Haycock et al., 2016).

To test the validity of the third assumption, multiple methods were employed. First, we calculated Cochran's Q-statistic to test for IV heterogeneity, which is indicated by a significant finding (*p* < 0.05). Heterogeneity can have several causes, of which horizontal pleiotropy is the most likely (Greco et al., 2015). To specifically address directional unbalanced pleiotropy, we performed MR‒Egger regression and MR-PRESSO (Mendelian Randomization Pleiotropy RESidual Sum and Outlier) analysis (Verbanck, Chen, Neale, & Do, 2018). Importantly, a previous simulation study showed that MR-PRESSO is more sensitive to horizontal pleiotropy than Egger's intercept (Verbanck et al., 2018).

Considering it unlikely that all IVs in MR fulfill the above outlined assumptions, several robust MR methods have been developed that differ in their robustness to violations of the MR assumptions (for details on the different methods, see the online SM1 – Supplementary Methods 6). Since no single method provides infallible proof of causality, using different methods has been recommended (Burgess et al., 2019) and is implemented in the present study. If Cochran's *Q* statistic suggested heterogeneity of the IV, the contamination mixture method and the MR-Lasso method were applied in addition to the penalized weighted median method.

For visualization of the results, Forest, scatter, funnel, and leave-one-out plots were created (see online SM1 – Supplementary Methods 6 and Figs S2–S4).

Power analysis to estimate the probability of finding a true effect was implemented using the sample size of outcome GWAS, the proportion of cases in the study, and the proportion of variance explained (*h*^2^_SNP_) by SNPs in the IV. Because SNP heritability was not reported by Hollis et al. (2020) for MPT, we estimated the explained variance in MPT by the 71 SNPs constituting our IV using the formula by Shim et al. (2015).

### Multivariable MR analysis – assessing the effect of BMI and other causes of pleiotropy

To consider BMI as a confounder, we performed multivariable MR with four different methods (for details on the different methods, see the online SM1 – Supplementary Methods 6), including multivariable MR-PRESSO. If there was no evidence of pleiotropy by multivariable MR-PRESSO, this implied that no further confounders needed consideration. Uncorrected effect sizes from MR-PRESSO correspond to those from the IVW method and will therefore not be reported. We further calculated the *Q*_a_-statistic to test for heterogeneity. Consistent with the univariate approach, a *p* value < 0.05 indicates heterogeneity in the multivariable MR model (Sanderson, Spiller, & Bowden, 2020). As individual genetic data were not available, we could not consider correlations between the exposure variables.

### Male pubertal timing

To construct the instrument variable, we used the genome-wide significant independent SNPs (*n* = 76) from the GWAS by Hollis et al. (2020). An effective GWAS meta-analysis sample size of 205,354 men of European ancestry was achieved by a multi-trait analysis of GWAS (MTAG) for continuous age at voice by data obtained from the UK Biobank and 23andMe study. MPT was constructed from the questions ‘When did your voice break?’ and ‘When did you start to grow facial hair’ (younger than average, about average, older than average, do not know, prefer not to answer). In the 23andMe study, the ‘age at voice breaking’ phenotype was determined by response to the question ‘How old were you when your voice began to crack/deepen?’. Participants could choose from seven predefined age bins, which were rescaled to one-year age bins (for further details on the GWAS, see the online SM1 – Supplementary Methods 7).

### Major depressive disorder

The data source for the outcome variable MDD was the recent GWAS by Howard et al. (2019) including 807,553 individuals (246, 363 cases and 561,190 controls). This meta-analysis was based on data from the three largest GWASs of depression. These studies used different measures for depression: (a) self-reported clinical diagnosis of MDD by Hyde et al. (2016); (b) MDD obtained from a structured clinical interview or based on broader criteria by Wray et al. (2018); (c) self-reported help-seeking for problems with nerves, anxiety, tension or depression (broad depression) by Howard et al. (2019). Overlapping samples were excluded. The meta-analysis identified 102 independent genome-wide significant variants. The genome-wide SNP-based heritability (*h*^2^_SNP_) was 8.9%. The proportion of females was 48% in Hyde et al. (2016) and 54% in Howard et al. (2019). The proportion of males and females was not reported by Wray et al. (2018) (for further details on the GWAS, see the online SM1 – Supplementary Methods 6).

### BMI

We used the most recent GWAS on BMI with sex-specific analyses (Pulit et al., 2019). A total of 806 ,834 individuals of European ancestry were included in the analysis, 374,756 of whom were males. The SNP-based heritability (*h*^2^_SNP_) for males was 35% for all SNPs (Pulit et al., 2019).

### Reporting and software

In reporting our studies, we followed the STROBE and STROBE-MR recommendations (Skrivankova et al., 2021). Data handling and analyses were either performed with SPSS 28.0 (IBM Corp., Armonk, NY) and its Complex Samples^®^ procedures to account for the sample design, design-related effects, and attrition related to sociodemographic characteristics (Mauz et al., 2020) or the software ‘R’ (3.5.1 and 4.1.1) with the R-packages ‘TwoSampleMR’ (0.4.26, https://github.com/MRCIEU/TwoSampleMR) (Hemani et al., 2018), ‘Mendelian Randomization’ (0.5.1; https://CRAN.R-project.org/package=MendelianRandomization) (Broadbent et al., 2020), ‘MVMR’ (0.3; https://github.com/WSpiller/MVMR) (Sanderson et al., 2020), and ‘MRPracticals’ (0.0.1; https://github.com/WSpiller/MRPracticals). MR-PRESSO was performed with R-package (1.0; https://github.com/rondolab/MR-PRESSO) (Verbanck et al., 2018).

## Results

### Epidemiological study – results

Of the 17,640 children and adolescents who took part in the baseline assessment of the KiGGS study, 2,732 were male and were queried on MDD history at wave 2. Of these, 2,026 participants were considered for further analyses regarding the cross-sectional analysis, as 706 were excluded for reasons outlined in Fig. 1.

### Missingness

Only regarding the history of MDD (unweighted: 3.1%, weighted 5.2%), MPT (weighted 13.7%, unweighted: 10.6%), and adverse life events (weighted 9.9%, unweighted: 9.0%) was there a significant (weighted) proportion (> 5%) of participants with missing information. Further analyses showed a random pattern of missingness.

Excluded participants were found to have a lower SES (*p* < 0.001), lower levels of social support (*p* = 0.004), lower self-efficacy (*p* = 0.02), and a history of more emotional childhood trauma (*p* = 0.04). However, absolute differences were minor (online SM1 – Supplementary Table S1), and effect sizes were either borderline small (SES: *d* = 0.22) or negligible (*d* < 0.20).

### Descriptives

**Figure 2.**
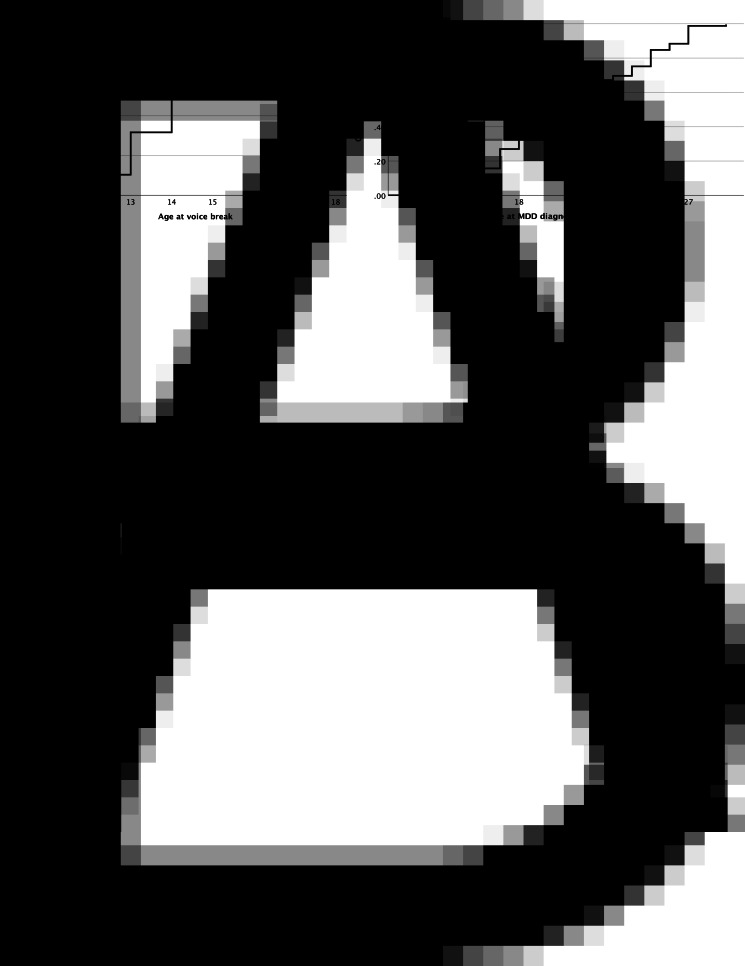
Cumulative probability of voice break (Panel A) and major depressive disorder (MDD, Panel B) dependent on age.

The mean age of participants at wave 2 was 23.93 (s.d. 3.35) years, and the mean age at voice break was 13.9 (s.d. 4.5) years (Table 1, Fig. 2A). The mean difference between the age at wave 2 and the age at voice break was 9.6 (s.d. 3.7) years. Considering a significant (*p* = 0.01) but trivial effect (*d* = 0.14) regarding the relationship between age at wave 2 and the reported age at voice break, no recall bias was evident.

**Table 1.** Descriptives

	Total (*N* = 2,026)	NO MDD (*N* = 1,914)	MDD (*N* = 112)
Age – KiGGS2	23.9 (3.4) [18.0–31.1]	23.8 (3.4) [18.0–31.1]	25.2 (3.1) [18.2–30.3]
BMI^a^	24.8 (4.5) [15.4–53.9]	24.8 (4.4) [15.4–53.9]	26.1 (5.6) [17.4–46.0]
Age at voice break	13.9 (1.3) [11–18]	13.9 (1.3) [11–18]	13.8 (1.5) [11–18]
Prevalence MDD	112 (6.9%)	–	112 (6.9%)
Age at MDD diagnosis	20.4 (4.3) [12–29]	–	20.4 (4.30) [12–29]
SES – MacArthur scale	5.5 (1.7) [1–10]	5.6 (1.60) [1–10]	4.4 (1.0) [1–8]
Social support	84.0 (17.6) [0–100]	84.7 (16.9) [0–100]	74.3 (23.4) [13–100]
Self-efficacy	68.6 (13.6) [17–100]	69.4 (13.2) [17–100]	58.4 (15.2) [20–100]
CTQ – emotional	8.0 (3.6) [2–25]	7.8 (3.3) [2–25]	10.5 (5.2) [4–25]
CTQ – physical	6.3 (1.9) [1–17]	6.2 (1.8) [1–17]	7.5 (2.8) [4–17]
Adverse life events	1.3 (1.2) [0–5]	1.3 (1.1) [0–5]	1.5 (1.3) [0–5]
Body self-image			
Much too thin	54 (2.1%)	49 (2.1%)	5 (3.3%)
A bit too thin	386 (17.1%)	367 (17.4%)	19 (13.6%)
Exactly the right weight	769 (37.5%)	741 (38.1%)	28 (28.8%)
Too obese	725 (37.3%)	674 (37.2%)	51 (38.1%)
Much too obese	92 (6.0)	83 (5.2%)	9 (16.3%)
ISCED			
1	2 (0.2%)	2 (0.2%)	–
2	307 (17.3%)	275 (15.7%)	32 (38.0%)
3	1007 (47.0%)	963 (47.8%)	44 (35.5%)
4	264 (13.8%)	250 (15.7%)	14 (11.8%)
5	–	–	–
6	308 (15.2%)	294 (15.7%)	14 (9.1%)
7	88 (4.4%)	83 (4.5%)	5 (3.9%)
8	5 (0.2%)	5 (0.3%)	-
Migration background (yes)	193 (13.8%)	185 (14.2%)	8 (8.7%)

MDD, major depressive disorder; SES, socioeconomic status; CTQ, childhood trauma questionnaire; ISCED, International Standard Classification of Education (1: primary education; 2: lower secondary education; 3: upper secondary education; 4: post-secondary non-tertiary education; 5: short-cycle tertiary education; 6: Bachelor's or equivalent level; 7: Master's or equivalent level; 8: Doctoral's or equivalent level).

Mean, standard deviation (in round brackets), and range (in square brackets) for interval scaled variables, absolute numbers and percentages otherwise.

*Note*: ISCED level (*N*_missing_ = 45) and migration background (*N*_missing_ = 3) were not part of the analyses but included for a more complete picture of demographic characteristics. Percentages are adjusted for the sampling plan, standard deviations are based on normalized weights. a = BMI is based on self-reported height and weight.

Over a median follow-up period of 11 years (IQR: 10–14), 112 cases of MDD were reported, of which only 25.9% were diagnosed before the age of 18 (Fig. 2B).

### Logistic regression results

Neither in the unadjusted (*b* = −0.05, *t* = −0.46, OR 0.95, 95%-CI [0.76–1.19], *p* = 0.65) nor the adjusted (*b* = 0.01, *t* = 0.06, OR 1.01, 95%-CI [0.81–1.26], *p* = 0.93) model considering important confounders in the relationship between MPT and MDD risk was there a significant finding in this regard (online Supplementary Material 2 (SM2) – Supplementary Table S2 for detailed results). This did not change when additionally considering interactions between MPT and confounders (*b* = 1.12, *t* = 1.08, OR 3.07, 95%-CI [0.40–23.74], *p* = 0.28). Power (1-*β*) to detect a borderline small effect size (*d* ⩾ 0.2) was 0.95. These findings were confirmed by the pooled analysis of imputed datasets (online SM2 – Supplementary Table 3). Since there was no evidence of a relationship between MPT and MDD, no mediation analysis was performed.

There was no evidence of a curvilinear relationship between MPT and MDD by the Box-Tidwell approach in either model.

### Mendelian randomization – results

For the univariable MR on the effect of MPT on MDD risk, 71 SNPs could be considered for the IV (online SM2 – Supplementary Table S4). The *F* statistic for the IV was 16 685.2. The *F* statistic for each SNP individually indicated that all SNPs were sufficiently strong instruments (lowest *F*-value = 29.9, online SM2 – Supplementary Table S5).

Cochran's *Q* test indicated heterogeneity of the IV (MR-Egger – *Q*(df = 69) = 186.4, *p* = 9.5 × 10^−13^; IVW – Q(df = 70) = 187.0, *p* = 1.3 × 10^−12^). Consistently, MR-PRESSO indicated pleiotropy even though Egger's intercept did not ( = −0.001, s.e. = 0.003, *p* = 0.66), likely for the reasons outlined above.

**Figure 3.**
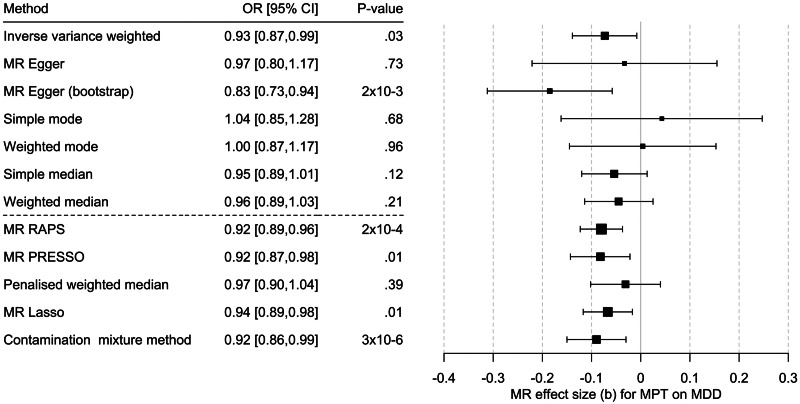
Results of the multi-SNP univariate Mendelian randomization (MR) analyses regarding the effect of male pubertal timing (MPT) [Hollis et al. (2020)] on MDD risk [Howard et al. (2019)]. OR = odds ratio, CI, confidence interval; b, unstandardized causal estimate of the change in risk for depression per one-year change in puberty time.

Univariable MR showed mixed results: IVW and MR-Egger with bootstrapping showed a significant causal effect of MBT on MDD. The methods robust to pleiotropy (MR RAPS, outlier corrected MR-PRESSO) and two of three methods robust to heterogeneity (contamination mixture method, MR Lasso) also showed a significant effect (Fig. 3, online SM2 – Supplementary Table S6 and Supplementary Figs S2–S4). However, the multivariable MR of the effect of MPT on MDD risk (*n* = 71; online SM2 – Supplementary Table 7), adjusted for BMI, showed that the effect observed in the univariable MR could be due to BMI-related pleiotropy (online SM1 – Supplementary Table S8 and Fig. S4), as none of the three multivariable methods showed an effect of MPT on MDD risk. Multivariate MR-PRESSO did not identify pleiotropic outliers. The conditional *F* test showed that the IV was sufficiently strong for both MPT (*F*_TS_ = 39.3) and BMI (*F*_TS_ = 27.0). The *Q*_a_ statistic indicated heterogeneity of the IV (*Q*[df = 68] = 180.6; *p* = 3.7 × 10^−12^).

### Power analysis

The *h*^2^_SNP_ for the 71 SNPs associated with MPT was 2.1% (online Supplementary Table S4). Our analysis had a power of 80% to detect an OR of 0.95 or 1.05 for MDD per one-year change in MPT and 100% power to detect an OR of 0.92 or 1.08 (online SM1 – Supplementary Fig. S1).

## Discussion

**Figure 4.**
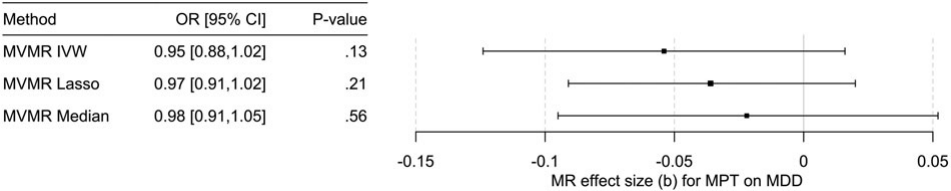
Results of the multivariable MR (MVMR) analyses of the causal effect of male pubertal timing (MPT) [Hollis et al. (2020)] on MDD risk [Howard et al. (2019)] adjusted for BMI [Pulit et al. (2019)] calculated using three different methods. OR, odds ratio; CI, confidence interval; b, unstandardized causal estimate of the change in risk for depression per one-year change in MPT.

Previous studies have provided inconsistent findings regarding the association of MPT with depressive symptoms and MDD (Hamlat et al., 2014), likely for methodological reasons. However, when investigating this relationship with a large epidemiological study considering multiple potential confounders and within the MR framework robust to residual confounding and reverse causation to consider the methodological limitations of each approach, there was no evidence of a (causal) relationship in this regard in the present work (Fig. 4).

### Male pubertal timing and MDD

The relationship between MPT and depression has mainly been studied dimensionally, that is, regarding the severity of depressive symptoms on a continuous scale. These studies have provided mixed evidence (Hamlat et al., 2014), reporting a relationship between depressive symptoms and either earlier or later pubertal timing, off-timing effects (i.e. effects of earlier and later pubertal timing), or no relationship. These inconsistencies also apply to those few studies addressing this relationship categorically, that is, the presence (or absence) of MDD according to clinical criteria, as investigated in the present study. While confounders were not considered in either study, Angold et al. (1998) found no evidence of a relationship between pubertal timing in girls and boys, but Hamlat et al. (2020) identified pubertal timing as a risk factor for incident and recurrent MDD in both sexes. However, considering the present study's findings, the latter study was likely affected by residual confounding. Moreover, this study may have also been insufficiently powered to conclusively investigate the impact of pubertal timing on MDD risk in boys, as discussed by the authors. In contrast, the present epidemiological analyses and the MR study were sufficiently powered for sound conclusions.

However, for the conclusions of the MR study to hold, the genetic architecture of depression should not change throughout the life course. As previously discussed in more detail (Hirtz et al., 2022b), a recent genome-wide association meta-analysis showed that the genetic architecture of internalizing symptoms is stable from early childhood to adolescence. This was concluded from the observation of overlapping SNP-based heritability estimates during earlier and later life and a high genetic correlation between childhood internalizing symptoms and adult depression (*r_g_* > 0.7) (Jami et al., 2022).

### Closely related but yet distinct – pubertal timing in girls and boys

The genetic SNP-based underpinnings of pubertal timing in females and males largely overlap (Hollis et al., 2020). Of the 76 SNPs related to MPT, 71 show a similar effect estimate of the same direction observed for age at menarche. This could imply that pubertal timing in both sexes shares the same underlying mechanisms to implicate sexual maturation in mental health. In females, downstream mechanisms to age at menarche have been suggested to explain its effect on MDD (Magnus et al., 2020), especially as there is no conclusive evidence that upstream mechanisms, including sex steroid hormones, are important in this regard (Hirtz et al., 2022b). Downstream mechanisms discussed include, for example, younger age at first delivery, an increased risk of childhood sexual abuse, and younger age at first sexual intercourse (Magnus et al., 2020). In particular, the latter is consistent with risky sexual behavior in adolescent boys with a small to medium effect size in a recent meta-analysis (Ullsperger & Nikolas, 2017) and a significant genetic correlation between MPT and health risk behaviors, including alcohol consumption and smoking (Hollis et al., 2020). However, at the same time, early MPT is associated with favorable social traits, including educational attainment (Hollis et al., 2020), which does not seem to apply to girls (Torvik et al., 2021). Moreover, physical changes related to pubertal maturation in boys are often considered desirable and convey social value, in contrast to adolescent girls (Rudolph & Troop-Gordon, 2010). Thus, adverse outcomes regarding mental health conferred by the genotype underlying pubertal timing might be counterbalanced by advantageous effects related to the same set of genes and their individual allelic architecture in males but not females. This explains the findings of the present study as well as the observation of adverse effects of earlier pubertal timing in girls on the mental health phenotype (Hirtz et al., 2022b).

However, the present study also provides insights into the underlying mechanisms relating pubertal timing to the etiology of MDD in boys. From a genetic perspective, BMI drives the apparent relationship between MPT and MDD due to an overlapping genetic origin of both exposure phenotypes. This is indicated by the multivariable MR analysis and the observation of no cause of horizontal pleiotropy other than via BMI. In this regard, BMI has not only been related to pubertal timing by epidemiological and MR studies (Busch et al., 2020) but has also been implicated in MDD (Casanova et al., 2021). BMI may exert its effects on MDD risk via several downstream outcomes, including, for example, hormone levels (Milano et al., 2020), peer relations (Kanders, Nilsson, & Åslund, 2021), and body image (Richard, Rohrmann, Lohse, & Eichholzer, 2016). In contrast, in the epidemiological analyses in the present study, neither BMI nor body image was related to MDD risk. However, since MR studies assess the average effect of an exposure over the life course, the direct and indirect effects of BMI may no longer be apparent or have yet to manifest in an epidemiological context.

### Limitations – epidemiological study

The age at voice break was self-reported in the epidemiological (and genetic) study. However, there was no evidence of a substantial recall bias, as far as can be inferred from our analyses, and the follow-up period in the present study was notably shorter than in previous studies reporting such a bias concerning pubertal timing (Must et al., 2002). Moreover, self-assessed age at voice break has been shown to correlate closely with other important pubertal events, justifying its use in epidemiological studies (Busch et al., 2019).

In neither the epidemiological analyses nor the MR study, we could consider subclinical depressive symptoms. As a consequence, our conclusions are limited to overt depression. Moreover, our study was limited to the analysis of the relationship between pubertal timing but not pubertal status and MDD. However, regarding subclinical depressive symptoms and pubertal status (Stumper & Alloy, 2023), previous studies have provided conflicting findings, which are suggestive of residual confounding, such as the analysis of pubertal timing. Thus, once data are available, it would be tempting to test for this by an MR study.

Moreover, confounders other than those included in the epidemiological analyses have been suggested but were not captured at either KiGGS assessment. However, issues of residual confounding are sufficiently covered by the MR analyses, confirming the results of the epidemiological studies. Also, neither migration nor social status is related to MPT in the KiGGS sample, rendering them unlikely confounders (Kahl, Schaffrath Rosario, & Schlaud, 2007).

### Limitations – MR study

By the statistical model of the GWAS by Hollis et al. (2020) underlying the MR analysis, we were limited to studying a linear relationship between MPT and MDD. However, there was no evidence of a relationship other than linear by the epidemiological study.

The analysis of the effect of BMI on MPT in the multivariable MR model relied on the GWAS by Pulit et al. (2019). While this GWAS allowed us to study sex-specific BMI-related findings pertaining only to males, it was conducted in adults. This implies a continuity of the genetic architecture of BMI over the life course regarding the present study, which is supported by a recent meta-analysis of 26 GWASs on childhood BMI. This study included data from 61 ,111 children and identified 25 childhood-specific BMI loci (Vogelezang et al., 2020). Most of these SNPs (20 of 25) were also related to adult BMI, and there was a genetic correlation between childhood and adult BMI of *r_g_* = 0.76.

The genetic instrument for depression was not sex specific, as no such information is available. However, as previously discussed (Hirtz et al., 2022b), a recent study that evaluated between-sex genetic heterogeneity in MDD using GWAS summary statistics from 29 cohorts found a genetic correlation close to one (Trzaskowski et al., 2019).

Moreover, our results apply to the Caucasian population only and likely do not generalize to patients with precocious puberty, even though SNPs in genes related to precocious puberty were used as IV as well (Hollis et al., 2020).

Adjusting for BMI could lead to an over-adjustment if BMI was a mediator. Thus, our approach provides a conservative estimate of the effect of pubertal timing on depression, recognizing that it might not capture the full direct effect. However, considering the KiGGS results which do not suggest a mediating effect of BMI on the relationship between MPT and MDD, our approach seems justified and highlights the advantage of the combined epidemiological and MR analysis.

Further minor methodological limitations are discussed in the online SM1 – Supplementary Discussion.

## Conclusions and implications

In contrast to females, our study shows that earlier MPT is not related to an increased MDD risk. This is suggested by the epidemiological and genetic analyses, combining methodological advantages of both approaches, which allows for sound conclusions.

## Supporting information

Hirtz et al. supplementary material 1 Hirtz et al. supplementary material

Hirtz et al. supplementary material 2 Hirtz et al. supplementary material

## Data Availability

The reported results related to the KiGGS study are based on a secondary analysis of data provided by the Robert Koch Institute (RKI), Germany. Requests to access the datasets should, therefore, be directed to the RKI (kiggsinfo@rki.de). Publicly available datasets analyzed in this study can be found here: https://www.ebi.ac.uk/gwas/. Original data generated and analyzed during this study (i.e. harmonized genetic data) can be found here: https://doi.org/10.6084/m9.figshare.19518982.v4.

## Supplementary material

The supplementary material for this article can be found at https://doi.org/10.1017/S0033291724000060
