# Supplementary material for: No relationship between male pubertal timing and depression – new insights from epidemiology and Mendelian randomization
Source: Psychol Med. 2024 Mar 22;54(9):1975–84. doi: 10.1017/S0033291724000060 (PMC11413349; doi:10.1017/S0033291724000060)
Supplement: Hirtz et al. supplementary material 1 — Hirtz et al. supplementary material [file S0033291724000060sup001.docx]

**Supplementary Material 1**

**No Relationship between Male Pubertal Timing and Depression – New Insights from Epidemiology and Mendelian Randomization**

Raphael Hirtz, Corinna Grasemann, Heike Hölling, Björn‑Hergen von Holt, Nicola Albers, Anke Hinney, Johannes Hebebrand, Triinu Peters

**Overview**

Supplementary Methods 1 – Details on the Sampling Method

Supplementary Methods 2 – Anthropometric Measures

Supplementary Methods 3 – Comparison of Descriptives and Analysis of Missingness

Supplementary Methods 4 – Confounders | Questionnaires

Supplementary Methods 5 – Testing of Statistical Assumptions

Supplementary Methods 6 – Mendelian Randomization Methods

Supplementary Methods 7 – GWAS Details (Male Pubertal Timing | Depression | BMI)

Supplementary Discussion – Limitations

Supplementary Table 1 – Descriptives Excluded Participants

Supplementary Table 6 – Results of the Univariable Mendelian Randomization Analyses

Supplementary Table 8 – Results of the Multivariable Mendelian Randomization (MVMR) Analyses

Supplementary Figure 1 – Power Analysis

Supplementary Figure 2 – Scatter Plot

Supplementary Figure 3 – Funnel Plot

Supplementary Figure 4 – Leave-One-Out Plot

Supplementary References

**Supplementary Methods 1** – Details on the Sampling Method

As previously detailed by Kurth, et al. 1, first, a systematic sample of 167 primary sample units (PSUs) was extracted from an inventory of German communities, which were stratified based on a classification system that gauges the degree of urbanization and geographic distribution. To determine the number of PSUs per stratum, a widely recognized method for community sampling was employed, where the sampling probability was proportional to the population size.

Secondly, for each birth cohort, an equal number of addresses (n = 24) were chosen at random from local population registries within the selected PSUs approximately 8 weeks before the beginning of the examinations. Depending on the PSU, the target population ranged from 144 to 180 individuals who were eligible for contact and invitation to the study. Recognizing a potential higher rate of unreachable contacts and non-responses from families of migrant nationality, a higher sampling rate was adopted for children and adolescents from these families compared to those from non-migrant families. The comprehensive sample from this approach comprised 28,299 children and adolescents.

Adhering to a predetermined route, 167 PSUs were assessed by four study teams over a span of three years (from 19 May 2003 to 6 May 2006). Multiple strategies were enacted to boost participation. Local media outlets and community networks announced the initiation of the survey in their respective communities. Direct communication was established with 14- to 17-year-olds and families who had not initially responded. Small incentives were also introduced as a means to encourage more participants. With the aim of enhancing participation from children of migrant backgrounds, communication materials including invitation letters and questionnaires were made available in six different languages. Additionally, outreach efforts were tailored to cater specifically to the unique migrant populace of each PSU.

**Supplementary Methods 2** – Anthropometric Measures

While all participants below 18 years of age were subjected to a physical examination at wave 2, height and weight was self-reported in 4,388 of 7,261 participants aged 18 years and older ^2^. Height was determined with a precision of 0.1 cm in upright posture using a calibrated stadiometer without wearing shoes. Weight was measured in underwear by an electronic scale displaying weight with a precision of 0.1 kg ^2^.

**Supplementary Methods 3** – Comparison of Descriptives and Analysis of Missingness

Participants with and without MDD were compared regarding important sociodemographic characteristics and covariates either by adjusted Wald F tests (continuous variables) or (second-order) adjusted χ^2^ tests according to Rao and Scott ^3^ (categorical variables). The pattern of weighted missing values was analyzed by separate variance t-tests and Little's test. When data was not missing completely at random (MCAR), multiple imputation was performed. Missing values were imputed in 5 datasets by the fully conditional specification (FCS) method as implemented in SPSS, using sample weights and the complete set of variables included in logistic regression model 2. For continuous variables, the range of imputed data was based on their observed distribution. Categorical variables were rounded to the nearest whole number, if applicable ^4^.

**Supplementary Methods 4** – Confounders | Questionnaires

*Childhood Trauma Questionnaire (CTQ)*

The CTQ assesses physical, emotional, and sexual abuse and neglect by 28 items. Each subscale consists of 5 items, rated on a 5-point Likert scale ranging from ‘not at all’ to ‘very frequently’. Higher scores indicate more neglect/abuse regarding the respective domain. Scores regarding each scale range between 5 (no abuse/neglect) to 25 (extreme abuse/neglect) points. Three questions additionally assess a tendency to trivialize or deny experiences of maltreatment. While the internal consistency (Cronbach’s α) of the scale ‚physical neglect‘ is weak, all other scales evidence high internal consistencies with α ≥ 0.8 ^5^. Moreover, construct validity has been supported by previous studies ^5^. In the present study, the subscales ‘physical abuse’ and ‘emotional abuse’ were used.

*WIRKALL-K Scale*

This scale evaluates general self-efficacy as the self-perceived ability to master difficult situations by 10 items scores on a 4-point Likert scale ranging from ‘not agree at all’ to ‘fully agree’. For the KiGGS study, scores were transformed to range between 0 (no self-perceived self-efficacy) and 100 (highly self-efficacious). The scale has good psychometric properties, with internal consistencies (Cronbach’s α) between .80 and .90 in the German norming sample ^2^.

*MacArthur Scale*

The MacArthur scales assesses the subjective social status by asking respondents by a single-scale item to place themselves on a 10-rung ‘social ladder’. This judgment is moderately to highly correlated with similar multidimensional indices of objective socioeconomic status, income level, occupational position, and educational attainment (r = 0.32−0.60) ^6^.

*Social Support Scale*

Eight items are used to determine the level of social support on a five-point scale (‘never’ to ‘always’). For the KiGGS study, scores were transformed to range between 0 (no support) and 100 (extensive support). The scale has good psychometric properties ^2,7^.

**Supplementary Methods 5** – Testing of Statistical Assumptions

The linearity of the relationship between continuous predictors and the logit transform of depression status was evaluated according to the Box-Tidwell approach. Variables of interest were screened for multicollinearity (>.70). All pairs of categorial variables were inspected for sufficient cell size (≥ 80% of cells with n ≥ 5).

**Supplementary Methods 6** – Mendelian Randomization Methods

**Univariable Methods**

As previously detailed ^8^, the following MR methods were used for this study: (a) the inverse-variance-weighted (IVW) method assumes that all ratio estimates provide independent evidence of the causal effect and that all genetic variants are valid instruments. No intercept term is included in the regression model ^9^. (b) MR–Egger considers an intercept term interpreted as the average pleiotropic effect of the genetic variants included in the analyses. If the pleiotropic effects are distributed independently of the genetic associations with the risk factor (InSIDE assumption: INstrument Strength Independent of Direct Effect), then the MR–Egger estimate is a consistent estimate of the causal effect as both the sample size and the number of genetic variants increase ^9,10^. (c) Mode-based estimation (MBE; simple mode, weighted mode) consistently estimates the true causal effect under the assumption that across all instruments, the most frequent value of bias due to pleiotropy is zero (ZEro-modal pleiotropy assumption (ZEMPA)) ^10^. Simple MBE is less accurate than weighted MBE, but simple MBE is less prone to bias due to violations of the InSIDE assumption. (d) Median-based estimators are consistent even when up to 50% of the instruments are invalid. The weighted median estimator has similar efficiency to the IVW method, but the simple median estimator is less efficient than either the IVW or the weighted median method ^11^. The penalized weighted median estimator is robust in the case of IV heterogeneity ^12^. (e) The robust adjusted profile score (MR RAPS) provides an overall estimator that is robust to systematic and idiosyncratic pleiotropy (some genetic instruments have a large effect on the outcome) ^13^. (f) MR-PRESSO uses a global bias test to assess whether the removal of potentially pleiotropic instruments results in a significant difference in the overall causal estimate. If so, a corrected causal estimate is calculated after removing the pleiotropic instruments ^14^. (g) The contamination mixture method identifies clusters of genetic variants with comparable causal estimates, which may represent different mechanisms by which the risk factor influences the outcome. It is robust and efficient in the presence of up to 40% invalid IVs ^15^; (h) MR-Lasso extends the IVW model to add an intercept term for each genetic variant. These intercept terms represent associations between genetic variants and the outcome that bypasses the risk factor. The causal effect is estimated by weighted linear regression, with the intercept terms subjected to lasso penalization. Lasso penalization shrinks the intercept of the valid instruments to zero ^12^. The MR Lasso method is carried out in two steps. First, a regularized regression model is fitted and a number of genetic variants are identified as valid instruments. Second, the causal effect is estimated using the standard IVW with only the valid genetic variants. If a large proportion of the variants are excluded as invalid, caution must be exercised in interpreting the results ^12^.

**Multivariable**

Four different methods to account for BMI as a covariate were employed ^16^: (a) The inverse variance method applies multivariable weighted linear regression. (b) Multivariable MR Lasso method adds an intercept term for each genetic variant to the multivariable IVW model. (c) The multivariable median method is similar to the univariable weighted median method, with the exception that it is based on quantile regression ^16^; (d) If multivariable MR-PRESSO ^14^ shows no evidence of pleiotropy, we will assume that no further covariates need to be considered.

**Plots**

To visualize the results of the single- and multi-SNP analyses, different plots were created. The scatter plots show single SNP effects on the exposure versus single SNP effects on the outcome with corresponding standard deviations and estimated regression lines of the multi-SNP analyses. Forest plot can be used to display MR estimates for individual SNPs and compare MR estimates from different methods of multi-SNP MR.

Funnel plots examine the relationship between study accuracy and effect size ^17^. Asymmetry in the funnel plot indicates a violation of MR assumptions ^18^. Whether a single SNP had a disproportionate effect on the regression coefficients can be assessed by a leave-one-out analysis within the IVW regression. In this analysis, the IVW regression is performed by omitting each genetic variant in turn ^9^.

**Supplementary Methods 7** – GWAS Details (Male Pubertal Timing | Depression | BMI)

**Male Pubertal Timing**

In the UK Biobank, relatively early and relatively late effect estimates were obtained using linear models which accounted for age, genotyping chip and ten principal components. In the 23andMe study, prticipants could choose from one of seven pre-defined age bins (under 9, 9–10 years old, 11–12 years old, 13–14 years old, 15–16 years old, 17–18 years old, 19 years old or older). These were then rescaled to one year age bins. Genetic associations with puberty timing were obtained by linear additive models using age and five principal components.

**Depression**

The regression model for the UK Biobank data included sex, age, genotyping array, and the first eight principal components for population structure ^19^. The regression model for the 23andMe data included the covariates age, sex, and the first five principal components ^20^.

**BMI**

The covariates included in the regression model for BMI are not reported ^21^.

**Supplementary Discussion** – Limitations

As previously mentioned, the covariance between AAM and BMI could not be considered in the multivariable MR analysis because individual data were not available. Two-sample MR assumes that the samples for the exposure and outcome are independent. However, a sample overlap of the GWAS on MPT with controls for the GWAS on MDD is theoretically possible. In a simulation analysis for binary outcomes with sample overlap in controls, there was no detectable bias in the IV estimates, even with extremely weak instruments, and no inflation of the type 1 error ^22^.

| **Supplementary Table 1** - Comparison of Included and Excluded Participants | | |
| --- | --- | --- |
|  | Included (N = 2,026) | Excluded |
| Age - KiGGS2 | 23.9 (3.4) [18.0 - 31.1] | 24.0 (3.4) [18.0 - 31.8] (N = 706) |
| BMI^a^ | 24.8 (4.5) [15.4 - 53.9] | 24.9 (4.7) [16.0 - 48.1] (N = 602) |
| Age at voice break | 13.9 (1.3) [11 - 18] | 14.1 (1.8) [10 - 23] (N = 416) |
| Prevalence MDD | 112 (6.9%) | 40 (7.2%) |
| Age at MDD diagnosis | 20.4 (4.3) [12 - 29] | 22.3 (5.1) [2 - 31] (N = 35) |
| SES - MacArthur scale* | 5.5 (1.7) [1 - 10] | 5.1 (1.8) [1 - 10] (N = 614) |
| Social support* | 84.0 (17.6) [0 - 100] | 80.3 (20.6) [9 - 100] (N = 693) |
| Self-efficacy* | 68.6 (13.6) [17 - 100] | 66.4 (16.1) [0 - 100] (N = 688) |
| CTQ - emotional* | 8.0 (3.6) [2 - 25] | 8.5 (3.9) [2 - 25] (N = 680) |
| CTQ - physical | 6.3 (1.9) [1 - 17] | 6.5 (2.5) [1 - 21] (N = 685) |
| Adverse life events | 1.3 (1.2) [0 - 5] | 1.2 (1.2) [0 - 5] (N = 461) |
| Body self-image |  |  |
| much too thin | 54 (2.1%) | 18 (2.3%) |
| a bit too thin | 386 (17.1%) | 137 (20.7%) |
| exactly the right weight | 769 (37.5%) | 259 (32.3%) |
| too obese | 725 (37.3%) | 220 (37.1%) |
| much too obese | 92 (6.0) | 46 (7.6%) |
| ISCED* |  |  |
| 1 | 2 (0.2%) | 5 (0.5%) |
| 2 | 307 (17.3%) | 151 (25.7%) |
| 3 | 1007 (47.0% | 324 (46.6%) |
| 4 | 264 (13.8%) | 72 (11.1%) |
| 5 | - | - |
| 6 | 308 (15.2%) | 90 (13.3%) |
| 7 | 88 (4.4%) | 17 (2.6%) |
| 8 | 5 (0.2%) | 1 (0.2%) |
| Migration background (yes)* | 193 (13.8%) | 96 (21.6%) |
| Mean, standard deviation (in round brackets), and range (in square brackets) for interval scaled variables, absolute numbers and percentages otherwise. MDD = major depressive disorder, SES = socioeconomic status, CTQ = childhood trauma questionnaire. ISCED = International Standard Classification of Education (1: primary education; 2: lower secondary education; 3: upper secondary education; 4: post-secondary non-tertiary education; 5: short-cycle tertiary education; 6: Bachelor's or equivalent level; 7: Master's or equivalent level; 8: Doctoral's or equivalent level). Note: ISCED level (N_missing_ = 45) and migration background (N_missing_ = 3) were not part of the analyses but included for a more complete picture of demographic characteristics. Percentages are adjusted for the sampling plan, standard deviations are based on normalized weights. a = BMI is based on self-reported height and weight. *Significant difference between included and excluded participants. | | |

**Supplementary Table 5 –** Results of the Univariable Mendelian Randomization Analyses of the Causal Effect of Male Pubertal Timing [Hollis. et al. ^23^] on MDD [Howard. et al. ^19^] - (N_SNP_ = 71)

| Method | b | | | se | p-value | OR | | |
| --- | --- | --- | --- | --- | --- | --- | --- | --- |
|  | Point estimate | Lower 95% CI | Upper 95% CI |  |  | Point estimate | Lower 95% CI | Upper 95% CI |
| Inverse variance weighted | -0.07 | -0.14 | -0.008 | 0.03 | **.03** | 0.93 | 0.87 | 0.99 |
| MR Egger | -0.03 | -0.22 | 0.16 | 0.10 | .73 | 0.97 | 0.80 | 1.17 |
| MR Egger (bootstrap) | -0.19 | -0.31 | -0.06 | 0.07 | **.002** | 0.83 | 0.73 | 0.94 |
| Simple mode | 0.04 | -0.16 | 0.25 | 0.10 | .68 | 1.04 | 0.85 | 1.28 |
| Weighted mode | 0.004 | -0.15 | 0.15 | 0.08 | .96 | 1.01 | 0.87 | 1.17 |
| Simple median | -0.05 | -0.12 | 0.01 | 0.03 | .12 | 0.95 | 0.89 | 1.01 |
| Weighted median | -0.05 | -0.11 | 0.03 | 0.04 | .21 | 0.96 | 0.89 | 1.03 |
| Robust adjusted profile score (RAPS) | -0.08 | -0.12 | -0.04 | 0.02 | **2.4x10^-04^** | 0.92 | 0.89 | 0.96 |
| MR-PRESSO outlier corrected | -0.08 | -0.14 | -0.02 | 0.03 | **.01** | 0.92 | 0.87 | 0.98 |
| Penalized weighted median | -0.03 | -0.10 | 0.04 | 0.04 | .39 | 0.97 | 0.90 | 1.04 |
| Lasso | -0.07 | -0.12 | -0.02 | 0.03 | **.008** | 0.94 | 0.89 | 0.98 |
| Contamination mixture* | -0.09 | -0.15 | -0.03 | 0.03 | **3.2x10^-06^** | 0.92 | 0.85 | 0.99 |

*Standard deviation of invalid estimates, start value psi=5, CI_min_=-1, CI_max_=5, CI_Step_=0.001. Significant findings are printed in bold type.

**Supplementary Table 7** - Results of the Multivariable MR (MVMR) Analyses of the Causal Effect of Male Pubertal Timing (MPT) [Hollis, et al. ^23^] on MDD Risk [Howard, et al. ^19^] Adjusted for BMI [Pulit, et al. ^21^] - (N_SNP_ = 71).

| Method | b | | | SE | P-value |
| --- | --- | --- | --- | --- | --- |
|  | Point estimate | Lower  95% CI | Upper  95% CI |  |  |
|  | **MPT** | | | | |
| MVMR IVW | -0.05 | -0.12 | 0.02 | 0.03 | .13 |
| MVMR Lasso^a^ | -0.04 | -0.09 | 0.02 | 0.03 | .21 |
| MVMR Median^b^ | -0.02 | -0.10 | 0.05 | 0.04 | .56 |
|  | **BMI in males** | | | | |
| MVMR IVW | 0.09 | -0.03 | 0.22 | 0.07 | .15 |
| MVMR Lasso^a^ | 0.11 | 0.02 | 0.20 | 0.05 | **.02** |
| MVMR Median^b^ | 0.11 | 0.003 | 0.22 | 0.06 | **.04** |

^a^ Number of variants: 71; Number of valid instruments: 42; Tuning parameter: 0.1740937


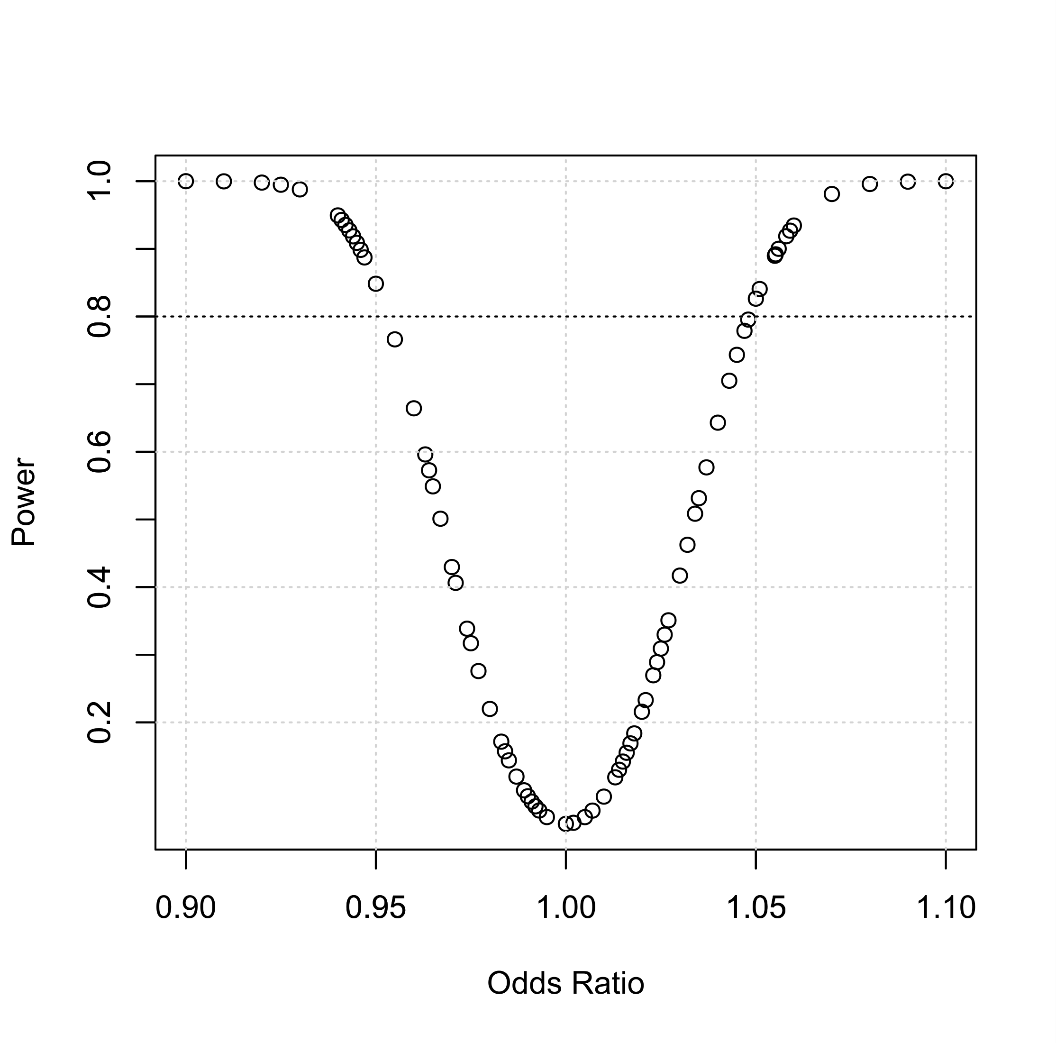


**Figure S1** - Results of the power analysis with N=71 SNPs for MPT. The dotted horizontal line corresponds to 80% power (OR=0.95 to OR=1.05). There was 100% power to detect OR=0.92 or 1.08 per one-year change in MPT.


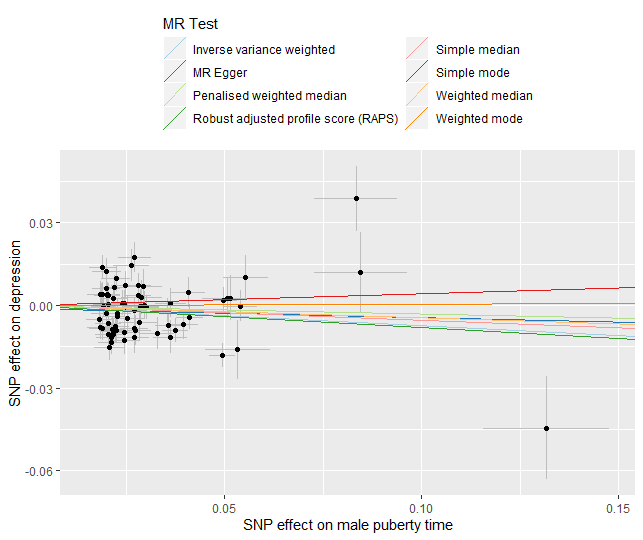


**Figure S2** - Scatter Plot of Genetic Associations With Male Pubertal Timing (Hollis et al., 2020) Against Risk for Depression (Howard et al., 2019) Using Different Mendelian Randomization (MR) Methods. The slopes of each line represent the causal association for each method.


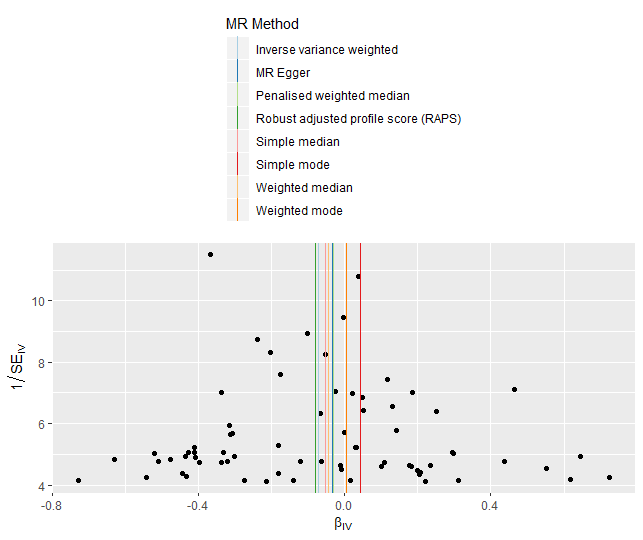


**Figure S3** - Funnel Plot: Mendelian Randomization (MR) Analyses With Male Pubertal Timing (Hollis et al., 2020) As Exposure and Risk for Depression (Howard et al., 2019) As Outcome. SE = standard error, IV = instrumental variable, β = unstandardized causal estimate of the change in risk for depression per one-year change in MPT


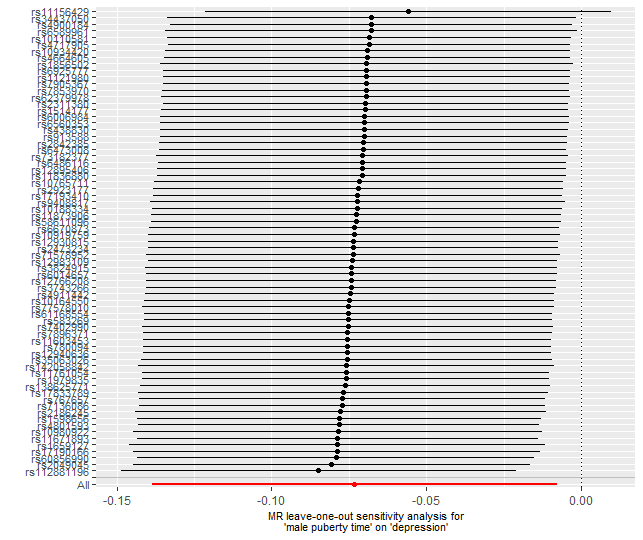


Figure S4 - Leave-One-out Analyses Using the IVW Method: Mendelian Randomization (MR) Analyses With Male Puberty Time (Hollis et al., 2020) As Exposure and Risk for Depression (Howard et al., 2019) As Outcome.

**Supplementary References**

1. Kurth BM, Kamtsiuris P, Holling H, et al. The challenge of comprehensively mapping children's health in a nation-wide health survey: design of the German KiGGS-Study. *BMC Public Health*. Jun 4 2008;8:196. doi:10.1186/1471-2458-8-196

2. Mauz E, Lange M, Houben R, et al. Cohort profile: KiGGS cohort longitudinal study on the health of children, adolescents and young adults in Germany. *Int J Epidemiol*. Apr 1 2020;49(2):375-375k. doi:10.1093/ije/dyz231

3. Rao JNK, Scott AJ. On Chi-Squared Tests for Multiway Contingency Tables with Cell Proportions Estimated from Survey Data. *Ann Stat*. 1984;12(1):46-60. doi:10.1214/aos/1176346391

4. Tabachnick BG, Fidell LS, Ullman JB. *Using multivariate statistics*. vol 5. Pearson Boston, MA; 2007:966.

5. Klinitzke G, Romppel M, Hauser W, Brahler E, Glaesmer H. [The German Version of the Childhood Trauma Questionnaire (CTQ): psychometric characteristics in a representative sample of the general population]. *Psychother Psychosom Med Psychol*. Feb 2012;62(2):47-51. Die deutsche Version des Childhood Trauma Questionnaire (CTQ) - psychometrische Eigenschaften in einer bevolkerungsreprasentativen Stichprobe. doi:10.1055/s-0031-1295495

6. Hoebel J, Muters S, Kuntz B, Lange C, Lampert T. [Measuring subjective social status in health research with a German version of the MacArthur Scale]. *Bundesgesundheitsblatt Gesundheitsforschung Gesundheitsschutz*. Jul 2015;58(7):749-57. Messung des subjektiven sozialen Status in der Gesundheitsforschung mit einer deutschen Version der MacArthur Scale. doi:10.1007/s00103-015-2166-x

7. Donald CA, Ware JE. The measurement of social support. *Research in Community & Mental Health*. 1984;4:325-370.

8. Hirtz R, Hars C, Naaresh R, et al. Causal Effect of Age at Menarche on the Risk for Depression: Results From a Two-Sample Multivariable Mendelian Randomization Study. *Front Genet*. 2022;13:918584. doi:10.3389/fgene.2022.918584

9. Burgess S, Thompson SG. Interpreting findings from Mendelian randomization using the MR-Egger method. *Eur J Epidemiol*. May 2017;32(5):377-389. doi:10.1007/s10654-017-0255-x

10. Hartwig FP, Davey Smith G, Bowden J. Robust inference in summary data Mendelian randomization via the zero modal pleiotropy assumption. *International journal of epidemiology*. Dec 1 2017;46(6):1985-1998. doi:10.1093/ije/dyx102

11. Bowden J, Davey Smith G, Haycock PC, Burgess S. Consistent Estimation in Mendelian Randomization with Some Invalid Instruments Using a Weighted Median Estimator. *Genetic epidemiology*. May 2016;40(4):304-14. doi:10.1002/gepi.21965

12. Rees JMB, Wood AM, Dudbridge F, Burgess S. Robust methods in Mendelian randomization via penalization of heterogeneous causal estimates. *PLoS One*. 2019;14(9):e0222362. doi:10.1371/journal.pone.0222362

13. Zhao Q, Wang J, Hemani G, Bowden J, Small DS. DAS IST DIE ALTE Statistical interference in two-sammple summary data Mendelian Randomization Robust Adjusted Profile Score. *arXiv:180109652 [statAP]*. 2019;

14. Verbanck M, Chen CY, Neale B, Do R. Detection of widespread horizontal pleiotropy in causal relationships inferred from Mendelian randomization between complex traits and diseases. *Nat Genet*. May 2018;50(5):693-698. doi:10.1038/s41588-018-0099-7

15. Burgess S, Foley CN, Allara E, Staley JR, Howson JMM. A robust and efficient method for Mendelian randomization with hundreds of genetic variants. *Nature communications*. Jan 17 2020;11(1):376. doi:10.1038/s41467-019-14156-4

16. Burgess S, Thompson SG. Multivariable Mendelian randomization: the use of pleiotropic genetic variants to estimate causal effects. *Am J Epidemiol*. Feb 15 2015;181(4):251-60. doi:10.1093/aje/kwu283

17. Haycock PC, Burgess S, Wade KH, Bowden J, Relton C, Davey Smith G. Best (but oft-forgotten) practices: the design, analysis, and interpretation of Mendelian randomization studies. *Am J Clin Nutr*. Apr 2016;103(4):965-78. doi:10.3945/ajcn.115.118216

18. Katikireddi SV, Green MJ, Taylor AE, Davey Smith G, Munafo MR. Assessing causal relationships using genetic proxies for exposures: an introduction to Mendelian randomization. *Addiction*. Apr 2018;113(4):764-774. doi:10.1111/add.14038

19. Howard DM, Adams MJ, Clarke TK, et al. Genome-wide meta-analysis of depression identifies 102 independent variants and highlights the importance of the prefrontal brain regions. *Nat Neurosci*. Mar 2019;22(3):343-352. doi:10.1038/s41593-018-0326-7

20. Hyde CL, Nagle MW, Tian C, et al. Identification of 15 genetic loci associated with risk of major depression in individuals of European descent. *Nat Genet*. Sep 2016;48(9):1031-6. doi:10.1038/ng.3623

21. Pulit SL, Stoneman C, Morris AP, et al. Meta-analysis of genome-wide association studies for body fat distribution in 694 649 individuals of European ancestry. *Hum Mol Genet*. Jan 1 2019;28(1):166-174. doi:10.1093/hmg/ddy327

22. Burgess S, Davies NM, Thompson SG. Bias due to participant overlap in two-sample Mendelian randomization. *Genet Epidemiol*. Nov 2016;40(7):597-608. doi:10.1002/gepi.21998

23. Hollis B, Day FR, Busch AS, et al. Genomic analysis of male puberty timing highlights shared genetic basis with hair colour and lifespan. *Nat Commun*. Mar 24 2020;11(1):1536. doi:10.1038/s41467-020-14451-5
